# Accelerated neuronal and synaptic maturation by BrainPhys medium increases Aβ secretion and alters Aβ peptide ratios from iPSC-derived cortical neurons

**DOI:** 10.1038/s41598-020-57516-7

**Published:** 2020-01-17

**Authors:** Tugce Munise Satir, Faisal Hayat Nazir, Dzeneta Vizlin-Hodzic, Erik Hardselius, Kaj Blennow, Selina Wray, Henrik Zetterberg, Lotta Agholme, Petra Bergström

**Affiliations:** 1 Institute of Neuroscience and Physiology, Department of Psychiatry and Neurochemistry, the Sahlgrenska Academy at the University of Gothenburg, S-405 30 Gothenburg, Sweden; 2 Institute of Neuroscience and Physiology, Department of Psychiatry and Neurochemistry, the Sahlgrenska Academy at the University of Gothenburg, S-431 80 Mölndal, Sweden; 3 Clinical Neurochemistry Laboratory, Sahlgrenska University Hospital, S-431 80 Mölndal, Sweden; 4 Department of Neurodegenerative Disease, Institute of Neurology, University College London Queen Square, London, WC1N 3BG UK; 5 UK Dementia Research Institute at UCL, London, WC1E 6BT UK

**Keywords:** Neurochemistry, Stem-cell differentiation, Neural stem cells, Cellular neuroscience, Molecular neuroscience

## Abstract

One of the neuropathological hallmarks of Alzheimer’s disease (AD) is cerebral deposition of amyloid plaques composed of amyloid β (Aβ) peptides and the cerebrospinal fluid concentrations of those peptides are used as a biomarker for AD. Mature induced pluripotent stem cell (iPSC)-derived cortical neurons secrete Aβ peptides in ratios comparable to those secreted to cerebrospinal fluid in human, however the protocol to achieve mature neurons is time consuming. In this study, we investigated if differentiation of neuroprogenitor cells (NPCs) in BrainPhys medium, previously reported to enhance synaptic function of neurons in culture, would accelerate neuronal maturation and, thus increase Aβ secretion as compared to the conventional neural maintenance medium. We found that NPCs cultured in BrainPhys displayed increased expression of markers for cortical deep-layer neurons, increased synaptic maturation and number of astroglial cells. This accelerated neuronal maturation was accompanied by increased APP processing, resulting in increased secretion of Aβ peptides and an increased Aβ38 to Aβ40 and Aβ42 ratio. However, during long-term culturing in BrainPhys, non-neuronal cells appeared and eventually took over the cultures. Taken together, BrainPhys culturing accelerated neuronal maturation and increased Aβ secretion from iPSC-derived cortical neurons, but changed the cellular composition of the cultures.

## Introduction

Amyloid plaques composed of aggregated amyloid beta (Aβ) peptides, predominantly species ending at amino acid 42 (Aβ42), are one of the major neuropathological hallmarks of Alzheimer’s disease (AD)^1^. Cerebrospinal fluid (CSF) Aβ42 concentration and the ratio of Aβ42 to Aβ40 in CSF are used as biomarkers for cerebral β-amyloidosis in AD^2^. Although some forms of these Aβ peptides are believed to be molecular triggers of AD, they are also produced and secreted by cells under normal physiological conditions^3–5^. Aβ peptides are generated by enzymatic cleavage of amyloid beta precursor protein (APP). Initially, APP is cleaved either by α-secretase, liberating soluble APPα (sAPPα), or by β-secretase, which releases sAPPβ. The remaining stub of APP in the latter processing pathway is then cleaved by γ-secretase to produce Aβ peptides of varying lengths^6^.

As an outcome of the discovery of induced pluripotent stem cells (iPSCs)^7^, functioning neurons of human origin can now be produced *in vitro* and these cells have also been shown by us and others to secrete measurable amounts of APP cleavage products into the cell media^8–10^. Moreover, ratios of short and long Aβ peptides (ranging in size from 14 to 42 amino acids) secreted into the cell media from these mature, human iPSC-derived neurons correspond to those measured in CSF^2,11^. There are many well-established, widely used protocols for cortical differentiation of human iPSCs. The one used in this study mirrors the human cortical development *in vivo* and gives rise to synaptically active neurons^12^. However, the protocol is time-consuming, as it takes up to 90 days to obtain mature neurons.

Neuronal maintenance medium (NMM), essentially a 1:1 mix of Neurobasal and DMEM/F12 media with supplements, is a commonly used medium to provide cortical differentiation and to maintain neuronal survival^10,12–14^. However, this conventional neuronal medium does not support neuronal functions and may even impair synaptic activity^15^. To address this, a medium formulated to improve the electrophysiological and synaptic properties of neurons was developed and named BrainPhys^15^. This medium contains factors, such as BDNF and GDNF, to increase the proportion of synaptically active neurons^15^. Meanwhile, increased synaptic activity has been shown to favor the differentiation of neuroprogenitor cells (NPCs) into functional neurons^16^. Similarly, synaptic activity-mediated increase in BDNF secretion from mature neurons has been shown to enhance the neuronal differentiation of precursor cells co-cultured with mature neurons^17^. Hence, regulating signaling pathways and neuronal activity could be a potential way to accelerate neuronal differentiation and maturation^18^.

BrainPhys has previously been investigated extensively for its ability to promote synaptic activity. However, to the best of our knowledge, the effects of BrainPhys on the secretion of APP cleavage products following cortical differentiation of human iPSC-derived NPCs has not yet been evaluated. To determine if culturing iPSC-derived NPCs in BrainPhys would accelerate the differentiation towards functional cortical neurons and if this consequently would affect the secretion of APP cleavage products, we performed a comparative study where human iPSC-derived NPCs were differentiated into neurons in BrainPhys in parallel with NMM. We found that neuronal differentiation of NPCs for less than 35 days in BrainPhys increased neurite branching, as well as the expression of markers for deep-layer cortical neurons, synaptic activity and glial cells in the cultures. Along with this, BrainPhys medium increased secretion of all soluble cleavage forms of APP that were measured, but with a significantly increased sAPPβ/sAPPα ratio indicating increased β-cleavage of APP, as well as shift towards increased γ-cleavage at Aβ amino acid 38. After more than 35 days in BrainPhys non-neuronal cell types appeared and rapidly took over the cultures however shorter differentiation time was sufficient to obtain cortical neurons secreting sAPP and long forms of Aβ. In conclusion, long-term BrainPhys culturing accelerates the differentiation of NPCs towards functional cortical neurons, but at the expense of neuronal purity. Future studies will reveal the consequences of the observed increased β-cleavage and secretion of Aβ38.

## Results

### BrainPhys accelerates neuronal differentiation

**Figure 1:**
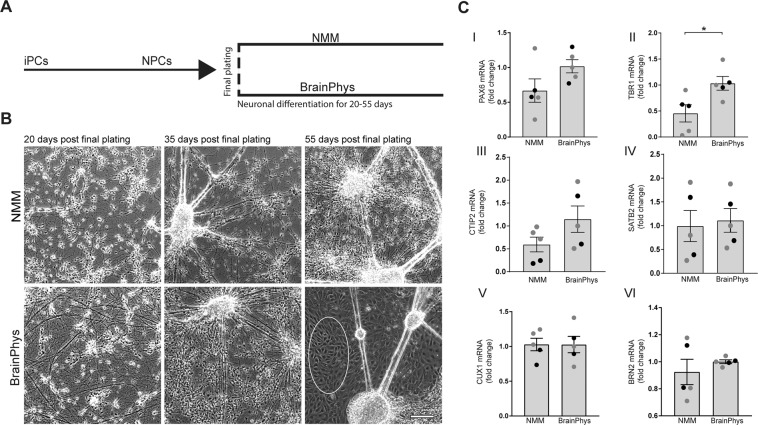
Differentiation of neuroprogenitor cells towards cortical neurons in neuronal maintenance- or BrainPhys medium. (**A**) Schematic illustration of the experimental setup. Human iPSCs are differentiated into neuroprogenitor cells (NPCs) according to Shi *et al*.^12^ up until final plating. Thereafter, the NPCs are further differentiated in NMM or BrainPhys for up to 55 days. (**B**) Representative phase-contrast images of the cultures 20, 35 and 55 days after final plating in NMM or BrainPhys medium. An increased neurite network is observed already after 20 days in BrainPhys (lower, left panel) as compared to NMM (upper, left panel). After 35 days in BrainPhys (lower, mid-panel), the cultures contain more neurons as compared to NMM (upper mid-panel), comparable to the cultures after 55 days of differentiation in NMM (upper, right panel). Extensive amounts of non-neuronal cells appear after 55 days in BrainPhys-medium (lower, right panel), marked with a white circle. Scale bar = 200 µm. **(C)** NPCs differentiated in BrainPhys or NMM for up to 35 days and investigated for changes in mRNA levels of markers for cortical layers measured with qPCR. (I) *PAX6* (paired-box transcription factor 6) mRNA, a marker for radial-glial progenitor cells, shows a trend to increase in BrainPhys-cultured cells, although not reaching statistical significance. (II) *TBR1* (T-Box Brain Protein 1) mRNA, a marker for cortical layer-VI neurons, increases significantly with BrainPhys. (III) *CTIP2* (B-cell lymphoma/leukemia 11B) mRNA, a marker for cortical layer-V neurons, shows a trend to increase in BrainPhys-cultured cells, although not reaching statistical significance. Upper-layer markers *SATB2* (Special AT-Rich Sequence-Binding Protein 2) (IV), *CUX1* (cut like homeobox 1) (V) and *BRN2* (Brain-Specific Homeobox/POU Domain Protein 2) (VI) mRNA levels do not differ between NMM- and BrainPhys-cultured cells. Mean values of five separate experiments on neurons from two different iPSC lines (Ctrl1 marked with grey circles and ChiPSC22 marked with black circles) were analysed with Student’s t-test. *p ≤ 0.05. Bars represent mean +/− SEM.

Human iPSCs were differentiated into NPCs according to a protocol by Shi *et al*.^12^ with minor changes^10^. This protocol robustly produces pure, cortical cultures in 90 days. Once the cells reached the NPC stage (after about 35 days of differentiation), final plating was performed and maturation was carried out in NMM or BrainPhys for another 20, 35 or 55 days (See Fig. 1A for experimental outline). During this process, cellular morphology was closely monitored and cortical identity and maturation were confirmed by analyzing mRNA and protein levels of markers of post-mitotic cortical neurons, synapses and astrocytes. At days 20 and 35 post final plating, a morphological examination revealed increased neurite networks in BrainPhys cultures as compared to NMM. After 35 days in BrainPhys, the cultures were morphologically similar to neurons cultured for 55 days in NMM (Fig. 1B). Beyond 35 days of culturing in BrainPhys, the presence of other non-neuronal cell types increased rapidly and hence subsequent analyses were performed on cells cultured in BrainPhys for a maximum of 35 days (a total differentiation time of 70 days). At this point, no statistically significant increase in the mRNA levels of *PAX6* (Fig. 1CI), a marker of radial glial progenitor cells, was observed, although they showed a tendency to increase in BrainPhys, while levels of *TBR1* mRNA (Fig. 1CII), a marker of cortical layer VI and post-mitotic projection neurons, increased significantly. The mRNA levels of *CTIP2* (Fig. 1CIII), a marker of cortical layer V neurons, also showed a tendency to increase in BrainPhys, while the mRNA levels of markers for upper-layer neurons, *SATB2, CUX1* and *BRN2* (Fig. 1CIV–VI), were unaffected.

### BrainPhys increases neurite branching and synaptic density

**Figure 2:**
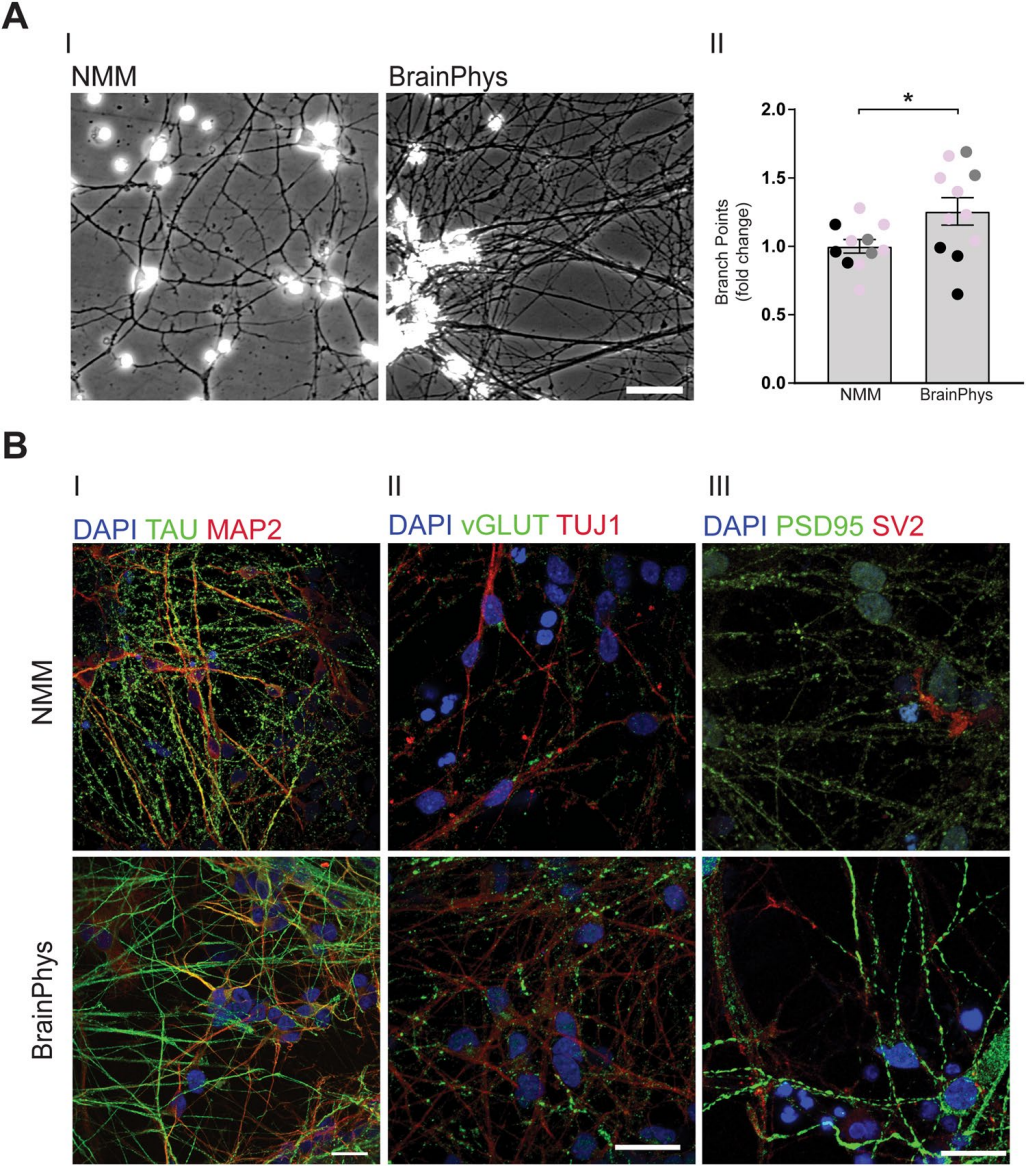
BrainPhys increases neuronal networks. NPCs were differentiated in BrainPhys or NMM for up to 35 days and investigated for changes in neuronal networks using bright-field imaging and immunocytochemistry. (**A**) BrainPhys increases neurite branching. (I) Representative bright-field images showing the neuronal networks in cells cultured in NMM (left panel) or BrainPhys (right panel). Scale bar = 20 µm. (II) Quantification of the number of neurite branch points shows an increase in cells cultured in BrainPhys already after ten days. Two-three images/experiment and condition from four separate experiments on neurons from three different iPSC lines (Ctrl1 marked with grey circles, ChiPSC22 marked with black circles and WTSIi015-A marked with pink circles) were analysed with Student’s t-test. *p ≤ 0.05. Bars represent mean +/− SEM presented as fold change relative to NMM. (**B**) BrainPhys increases markers of neuronal processes and synapses. Representative immunocytochemistry images of neuronal markers in NMM- and BrainPhys-cultured cells. (I) TAU (microtubule-associated protein tau, green): a marker of axonal microtubules and MAP2 (microtubule-associated protein 2, red): a marker for neuron-specific microtubuli of dendrites. (II) vGLUT1 (vesicular glutamate transporter 1, green): a synaptic marker for glutamatergic pre-synapses and TUJ1 (neuron-specific class III beta-tubulin, red): a marker for microtubule stability of axons. (III) PSD-95 (postsynaptic density protein 95, green): a marker of post-synaptic density and SV2 (synaptic vesicle protein 2, red): a marker of synaptic vesicles. Upper panel = NMM, lower panel = BrainPhys. Scale bar = 25 µm.

After ten days of culture in BrainPhys, we observed that the cells displayed more neurites as compared to NMM-cultured cells (Fig. 2AI). To examine if BrainPhys increased neurite branching, we used phase-contrast images from each population and quantified the number of neurite branch points per image related to growth area. The number of branch points in BrainPhys-cultured neurons was then related to the number of branch points in NMM-cultured neurons. BrainPhys-cultured cells displayed a significantly increase in branch points as compared to NMM-cultured cells (Fig. 2AII).

Next, we performed immunocytochemistry to examine the expression and localization of proteins specific to neurons in the two culture conditions. Cells differentiated in both media expressed all the investigated neuronal markers. Although both cell populations stained positive for the microtubule-associated proteins MAP2 and tau (Fig. 2BI), NMM-cultured cells (upper panels) displayed more punctuated tau-staining along the axons as compared to BrainPhys-cultured neurons (lower panels). The BrainPhys-cultured cells showed more neuron-specific Class III β-tubulin (TUJ1) positive axons and a higher density of vesicular glutamate transporter, vGLUT1, positive synapses (Fig. 2BII) as compared to the NMM-cultured cells. BrainPhys-cultured cells also displayed more staining of postsynaptic density protein 95 (PSD95), a post-synaptic protein, and synaptic vesicle protein 2 (SV2), a pre-synaptic protein (Fig. 2BIII) as compared to the NMM-cultured cells.

### BrainPhys increases synaptic activity and expression of synaptic markers

**Figure 3:**
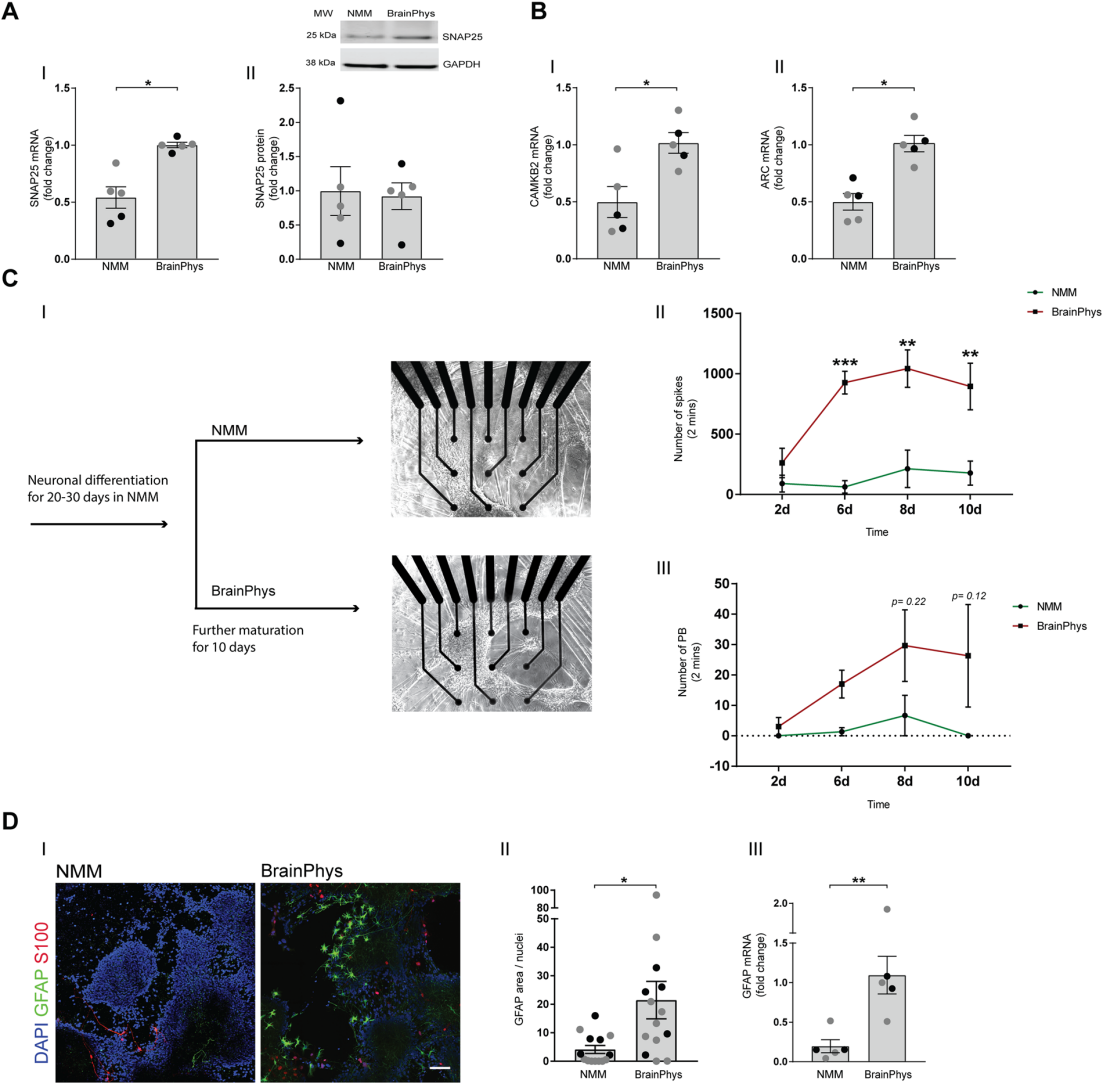
BrainPhys increases expression of neuronal activity markers and enhances neuronal activity. NPCs differentiated either in BrainPhys medium or in NMM for up to 35 days and investigated for changes in expression of markers for neuronal activity using qPCR and western blot. Neuronal activity investigated using multi electrode array (MEA) analysis, and expression of astrocyte markers investigated using immunocytochemistry and qPCR. (**A**) mRNA levels (I) of *SNAP25* (synaptosomal nerve-associated protein 25), a pre-synaptic protein also known to regulate dendritic spines, significantly increases in BrainPhys-cultured neurons, while SNAP25 protein levels (II) remain stable. The blot from one representative experiment is shown. Mean values of five separate experiments on neurons from two different iPSC lines (Ctrl1 marked with grey circles and ChiPSC22 marked with black circles) were analysed with Student’s t-test. *p ≤ 0.05. Bars represent mean +/− SEM. (**B**) mRNA levels of (I) *CAMK2B* (calcium/calmodulin-dependent protein kinase type II beta chain), a protein required for long-term potentiation (LTP), and (II) *ARC* (activity regulated cytoskeleton associated protein), a synaptic activity marker, increase significantly in BrainPhys-cultured neurons. Mean values of five separate experiments on neurons from two different iPSC lines (Ctrl1 marked with grey circles and ChiPSC22 marked with black circles) were analysed with Student’s t-test. *p ≤ 0.05. Bars represent mean +/− SEM. (**C**) Mature, NMM-differentiated neurons cultured in BrainPhys medium for ten days examined for changes in neuronal activity using multi-electrode assay (MEA) analysis (I). The number of spikes (II) increase significantly already after six days with BrainPhys and stay elevated over time. Although not statistically significant, (III) the number of population bursts (PB) shows a trend to increase in BrainPhys cultures as compared to NMM. (**D**) Representative immunocytochemistry images of the astroglial markers GFAP and S100 (I) show an increased staining in BrainPhys cultures. GFAP (glial fibrillary acidic protein, green): a specific marker for astrocytes, S100 (S100 calcium-binding protein, red): a marker for glial cells. Nuclei are stained with DAPI (blue). Scale bar = 100 µm. (II) The area of GFAP signal per image was quantified and related to the number of DAPI-stained nuclei in the same image. Three-six images/experiment and condition from three separate experiments on neurons from two different iPSC lines (Ctrl1 marked with grey circles and ChiPSC22 marked with black circles) were analysed with Student’s t-test. *p ≤ 0.05. Bars represent mean +/− SEM. (III) mRNA levels of *GFAP* increase significantly in BrainPhys cultures. Mean values of five separate experiments on neurons from two different iPSC lines (Ctrl1 marked with grey circles and ChiPSC22 marked with black circles) were analysed with Student’s t-test. **p ≤ 0.01. Bars represent mean +/− SEM.

To investigate if BrainPhys culturing would accelerate synaptic development, we next performed analysis of proteins known for their involvement in long-term memory formation and synaptic maturation. mRNA levels of *SNAP25*, a pre-synaptic protein known to be involved in neurotransmitter release, synaptic transmission and maturation of synaptic density^19,20^, increased significantly in BrainPhys-cultured cells (Fig. 3AI), whereas protein levels of SNAP25 did not change (Fig. 3AII). *CAMK2B* is known to have a role in synaptic plasticity^21^ as well as maintaining the structure of dendritic spines^22^ and *ARC* is one of the neuronal activation immediate-early genes (reviewed in^23^), the transcription of which has been shown to increase by neuronal activity^24^. Hence, both *ARC* and *CAMK2B* mRNA are used to measure changes in synaptic activity^25–27^. mRNA levels of both *CAMK2B* (Fig. 3BI) and *ARC* (Fig. 3BII) increased significantly in BrainPhys-cultured cells as compared to NMM. Together, this suggests that BrainPhys media renders a neuronal population with more mature synapses, in line with the observed increase in neurite branching.

Next, we investigated a possible effect of BrainPhys culturing on spontaneous neuronal activity by using Multi-Electrode Array (MEA). Final plating was performed in 6-well MEA plates with NMM. Twenty to thirty days after the final-plating, NMM was replaced by BrainPhys in half of the wells, and neuronal activity was recorded from each condition every second day for ten days. Due to neuronal migration, we controlled that the cells were on the electrodes just before recordings were performed (Fig. 3CI). Number of spikes (action potentials) and population bursts were used to determine changes in overall neuronal activity and network activity, respectively. Six days of culturing in BrainPhys was enough to significantly increase the number of spikes as compared to NMM (Fig. 3CII). The spikes in BrainPhys-cultured cells seemed more organized in bursts, although not reaching statistical significance (Fig. 3CIII).

### BrainPhys increases the number of glial cells

Since astrocytes are known to interact with neurons to regulate synaptic function (reviewed in^28^), we analyzed the astrocyte content in cultures using immunocytochemistry and qPCR. An increased staining of GFAP- and S100-positive astrocytes was observed in BrainPhys cultures compared with NMM (Fig. 3DI). Quantification of GFAP staining showed an increased GFAP area per cell (Fig. 3DII) in BrainPhys cultures, along with increased mRNA levels of *GFAP* (Fig. 3DIII**)**. This indicates that BrainPhys increases the astrocytic population during differentiation.

### BrainPhys increases expression and secretion of axonal proteins

**Figure 4:**
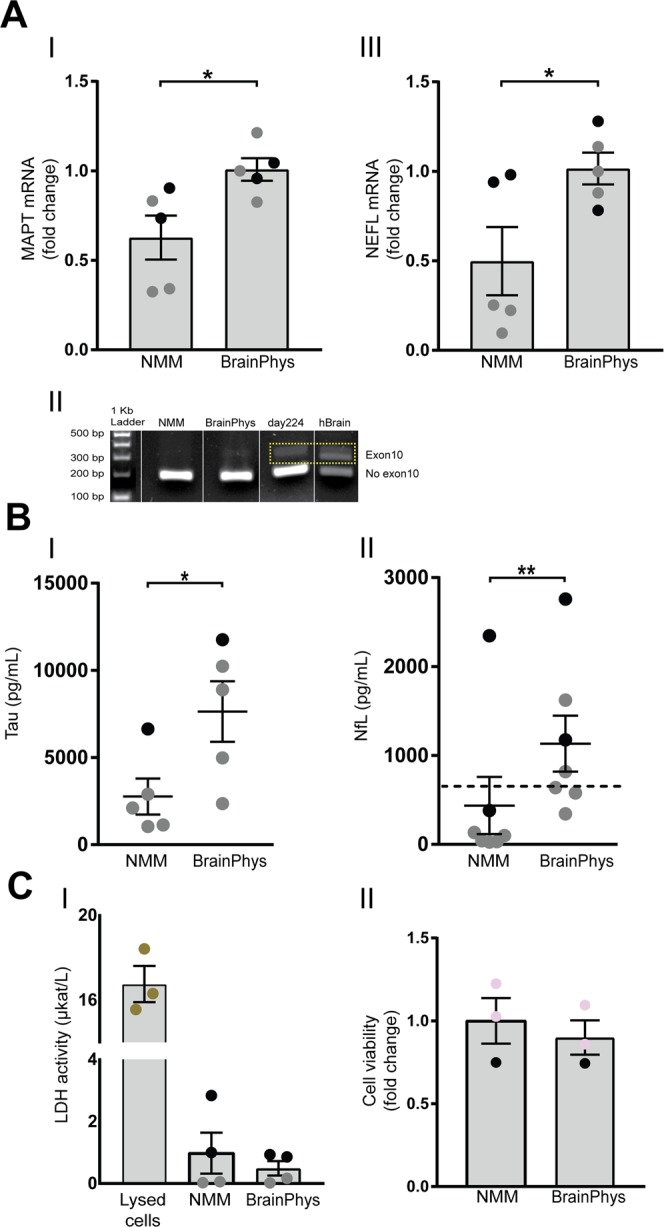
BrainPhys increases media concentrations of axonal proteins. NPCs differentiated in BrainPhys or NMM for up to 35 days and investigated for changes in mRNA expression and secretion of axonal proteins. (**A**) mRNA levels of *MAPT* (I), the gene coding for Tau, and *NEFL* (III), the gene coding for NfL, measured with qPCR both increase significantly in the BrainPhys-cultured cells. Mean values of five separate experiments on neurons from two different iPSC lines (Ctrl1 marked with grey circles and ChiPSC22 marked with black circles) were analysed with Student’s t-test. *p ≤ 0.05. Bars represent mean +/− SEM. (II) One representative gel comparing tau splice variants in NMM- and BrainPhys cultures shows that BrainPhys did not induce the mature form of tau including exon 10. Human whole brain cDNA and neurons differentiated in NMM for 224 days are included as positive controls. The whole gel image is shown in Supplementary Fig. 1F. (**B**) Concentrations of secreted Tau and NfL into the cell-conditioned media measured with ELISAs. Culturing the cells in BrainPhys significantly increases the concentration of Tau (I) and NfL (II) in the cell media, where the concentrations of NfL in the NMM-cultured cells is mostly under the detection limit of 50 pg/mL (dotted line). Tau: Mean values of five separate experiments on neurons from two different iPSC lines (Ctrl1 marked with grey circles and ChiPSC22 marked with black circles) were analysed with Student’s t-test. NfL: Mean values of six separate experiments on neurons from two different iPSC lines (Ctrl1 marked with grey circles and ChiPSC22 marked with black circles) were analysed with Student’s t-test. *p ≤ 0.05, **p ≤ 0.01. Lines represent mean +/− SEM. (**C**) (I) Released lactate dehydrogenase (LDH) into the cell-conditioned media, measured with LDH activity assay. No significant differences in LDH release is observed between cells cultured in NMM or BrainPhys. As a positive control, cells lysed in 1% (v/v) Triton-X100 to release all LDH is included. Mean values of four separate experiments on neurons from two different iPSC lines (Ctrl1 marked with grey circles and ChiPSC22 marked with black circles) were analysed with Student’s t-test. (II) Cell viability determined by measuring acridine orange-positive (total) cells and DAPI-positive (dead) cells, using image cytometry. No significant difference is observed between NMM and BrainPhys cultures. Mean values of three separate experiments on neurons from two different iPSC lines (WTSIi015-A marked with pink circles and ChiPSC22 marked with black circles) were analysed with Student’s t-test. Bars represent mean +/− SEM presented as fold change relative to NMM.

To investigate if the increase in neuronal branching was reflected in increased expression of microtubule-organizing proteins, we analyzed expression and secretion of tau, a stabilizing protein in axonal microtubules^29^, and neurofilament-light (NfL), a modulator of axonal caliber^30^. Culturing the cells in BrainPhys significantly increased the mRNA levels of *MAPT*, the gene coding for tau (Fig. 4AI). We next performed a PCR analysis to investigate if we could detect the mature splice form of tau including exon 10 which is present in the cortex of children and adults, but not in the fetal cortex^31^. The exon 10 splice variant could not be detected in either condition at this neuronal differentiation stage (Fig. 4AII, the full image of the gel is shown in Supplementary Fig. 1F). Neurons cultured in NMM for 224 days and cDNA from whole brain, where the exon 10 splice variant is present, were included as positive controls. mRNA levels of *NEFL*, the gene coding for NfL, also increased significantly as compared to NMM (Fig. 4AIII). To investigate if the increased mRNA levels were reflected in secretion of the corresponding proteins, concentrations of tau and NfL were measured in the cell-conditioned media using ELISA. BrainPhys culturing significantly increased the concentration of tau (Fig. 4BI) and the concentration of NfL from undetectable to clearly detectable levels (Fig. 4BII) in the cell-conditioned media, as compared to NMM cultures. To exclude the possibility that increased secretion of tau and NfL was due to leakage from dying cells, lactate dehydrogenase (LDH) was measured in the cell-conditioned media from NMM and BrainPhys cultures as a marker of cell-membrane permeability due to necrosis^32^. Suspensions from cells lysed with Triton X-100 were used as positive control. The LDH concentrations were generally low (5% and 3% of the positive control for NMM and BrainPhys, respectively) and there was no difference in LDH release from BrainPhys-cultured cells compared with NMM (Fig. 4CI). In addition, the viability of cells cultured in NMM or BrainPhys was assessed using image cytometry. No differences were observed in viability between the two cell culture conditions (Fig. 4CII**)**.

### BrainPhys changes APP processing

**Figure 5:**
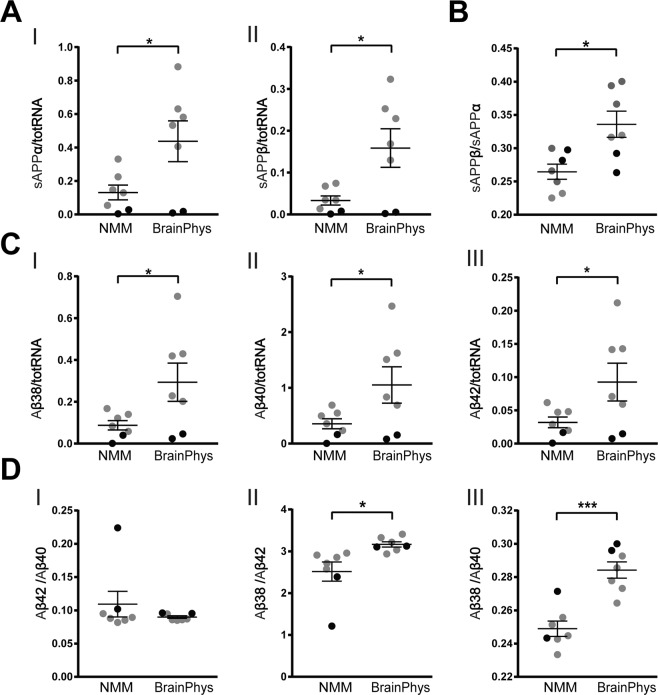
BrainPhys changes APP processing. NPCs differentiated in BrainPhys or NMM for up to 35 days and the concentrations of APP-cleavage products in the cell-conditioned media measured with immunochemiluminescence methods. Concentrations of secreted cleavage products of APP normalized to total RNA extracted from the corresponding cell lysate. (**A**) Concentrations of secreted sAPPα (I) and sAPPβ (II) measured in the cell-conditioned media and normalized to total RNA from the corresponding cell lysate, both significantly increase in BrainPhys-cultured neurons compared with NMM cultures. (**B**) The ratio of sAPPβ to sAPPα increases in BrainPhys-cultured cells compared with NMM cultures, indicating an increased β-site cleavage of APP. (**C**) Concentrations of secreted Aβ peptides were measured in the cell-conditioned media and normalized to total RNA from the corresponding cell lysate. Aβ38 (I), Aβ40 (II) and Aβ42 (III) all increase significantly with BrainPhys compared with NMM. (**D**) The ratios of Aβ42 to Aβ40 (I) does not differ between BrainPhys- and NMM cultures, whereas the ratio of Aβ38 to Aβ42 (II) and Aβ38 to Aβ40 (III) increase significantly. This indicates an increased secretion of Aβ38 over Aβ42 and Aβ40 in the BrainPhys-cultured cells. Mean values of seven separate experiments on neurons from two different iPSC lines (Ctrl1 marked with grey circles and ChiPSC22 marked with black circles) were analysed with Student’s t-test. *p ≤ 0.05, **p ≤ 0.01, ***p ≤ 0.001. Bars represent mean +/− SEM.

We have previously shown that secretion of APP cleavage products changes with neuronal and synaptic maturation^10^. Therefore, we next investigated the effects of BrainPhys culturing on neuronal secretion of APP cleavage products. The concentrations of sAPPα and sAPPβ, as well as Aβ38, Aβ40 and Aβ42, in cell-conditioned media were measured using immunoassays with electrochemiluminescence detection and normalized to total RNA from the same well (Fig. 5). Culturing neurons in BrainPhys significantly increased the secretion of both sAPPα and sAPPβ as compared to NMM-cultured cells (Fig. 5AI–II). Furthermore, the ratio of sAPPβ to sAPPα in the cell media was significantly higher in BrainPhys compared with NMM-cultured cells (Fig. 5B), indicating an increased β-cleavage of APP. Consistently, the media concentrations of Aβ38, Aβ40 and Aβ42 were all significantly increased in BrainPhys compared with NMM-cultured cells (Fig. 5CI–III).

The Aβ region of APP can be cleaved by γ-secretase at different amino acids and more cleavage at amino acid 42 than at 38 and 40 is likely the most important amyloidogenic effect of presenilin [PSEN; the active site of γ-secretase] mutations that cause familial AD^33^. Hence, to investigate if culturing in BrainPhys would also modulate γ-secretase-mediated cleavage at the C-terminus of Aβ, we calculated the ratios of Aβ42/Aβ40, Aβ38/Aβ40 and Aβ38/Aβ42. The ratio of Aβ42/Aβ40 did not change by BrainPhys-culturing (Fig. 5DI), while the Aβ38/Aβ40 and Aβ38/Aβ42 ratios were significantly higher in BrainPhys-cultured cells as compared to NMM (Fig. 5DII–III). This indicates that BrainPhys has an increasing effect on γ-secretase cleavage at amino acid 38 of Aβ (*i.e*., a cleavage pattern that could protect from amyloidosis)^33^.

### Blocking synaptic activity decreases Aβ secretion

**Figure 6:**
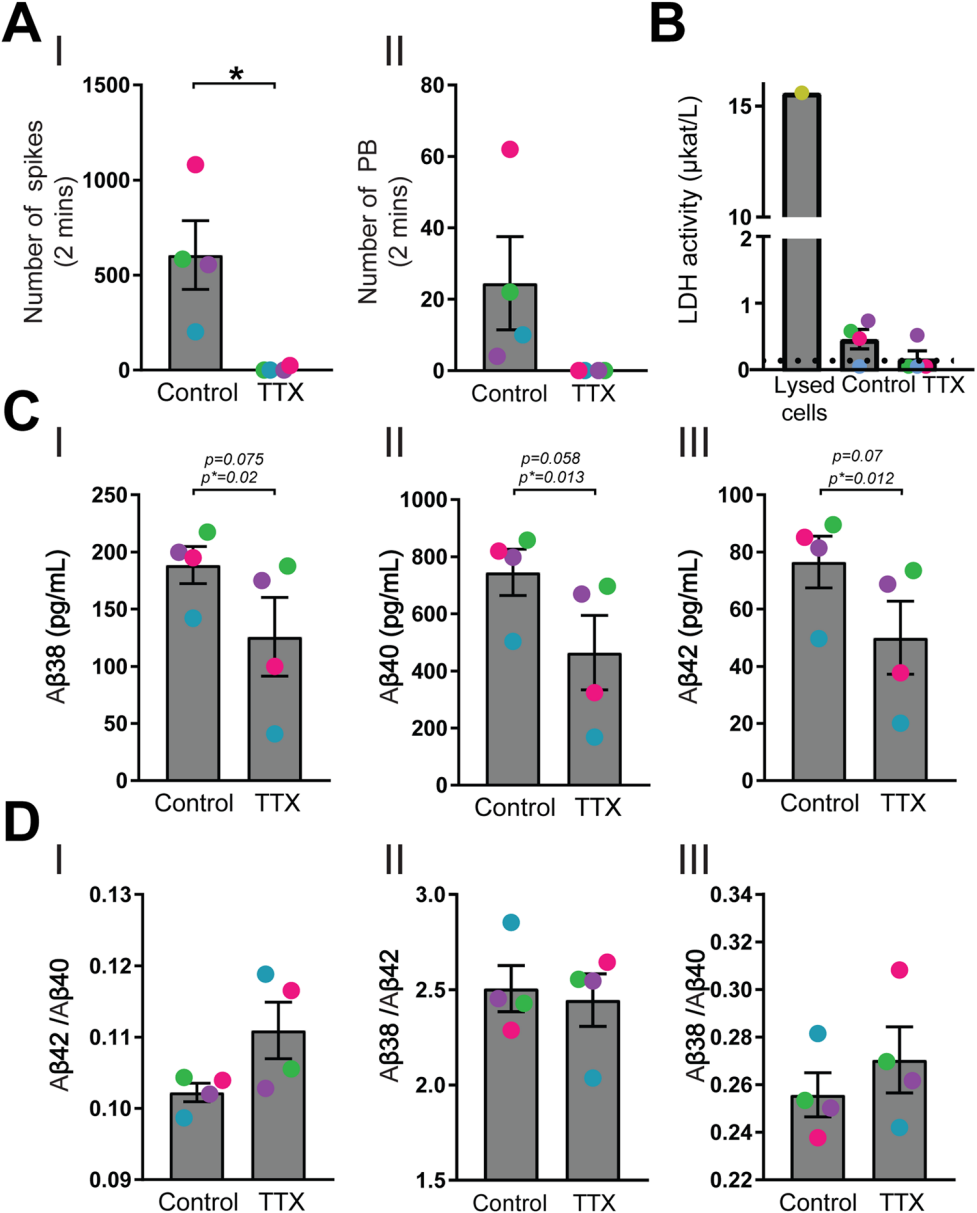
Tetrodotoxin blocks synaptic activity and decreases Aβ secretion. Neurons cultured in BrainPhys for ten days were treated with 1 µM tetrodotoxin (TTX) for 24 hours and the effects of synaptic activity on secreted Aβ peptides analyzed. (**A**) The number of spikes (I) and population bursts (II) are still decreased after 24 hours of TTX treatment, showing a prolonged blockage of synaptic activity with TTX. (**B**) Released lactate dehydrogenase (LDH) into the cell-conditioned media, measured with LDH activity assay on the cell media to exclude TTX-induced cell death. No significant differences in LDH release are observed between control- and TTX-treated neurons. As a positive control, cells lysed in 1% (v/v) Triton-X100 to release all LDH is included. Samples under the limit of detection of 0,17 µkat/L (shown with black dotted line), are plotted as 0.085 (half of the value for the limit of detection). (**C**) The secretion of Aβ38 (I), Aβ40 (II) and Aβ42 (III) decreases in all experiments with TTX, as compared to vehicle control. “p” indicates un-normalized p values whereas “p*” indicates p-value for data normalized to the control. (Each experiment is marked with separate colours to show the individual effect). (**D**) The ratios of Aβ42/40 (I), Aβ38/42 (II) and Aβ38/40 (III) do not change significantly by TTX treatment. Mean values of four separate experiments on neurons from two different iPSC lines (ChiPSC22 marked with pink circles and WTSIi015-A marked with green, blue and purple circles) were analysed with Student’s t-test. Bars represent mean +/− SEM. *p ≤ 0.05.

Tetrodotoxin (TTX) has previously been shown to block synaptic activity and to decrease Aβ secretion with approximately 25% *in vitro*^34^. To investigate if the increased Aβ secretion seen in BrainPhys-cultured cells could be explained by increased synaptic activity, neurons cultured in BrainPhys for ten days were treated with 1 µM TTX for 24 hours and compared to vehicle control. MEA recordings performed immediately before collection of the conditioned media showed that the cells were still synaptically inactive after 24 hours of TTX treatment (Fig. 6AI,II). To exclude that this effect was due to cell death, LDH was measured in the cell-conditioned media. No significant differences in LDH activity were observed between control- and TTX-treated neurons, indicating that 1 µM TTX for 24 hours did not induce cell death (Fig. 6B). The TTX treatment decreased the secretion of Aβ peptides compared with control in all four experiments (the individual experiments are marked with separate colors) (Fig. 6CI–III). We next investigated if blocking synaptic activity with TTX treatment would affect the ratios of the secreted peptides, but no consistent changes in the ratios of Aβ42/Aβ40, Aβ38/Aβ40 and Aβ38/Aβ42 were observed (Fig. 6DI–II**)**.

### BrainPhys increases expression of APP and APP-cleaving secretases

**Figure 7:**
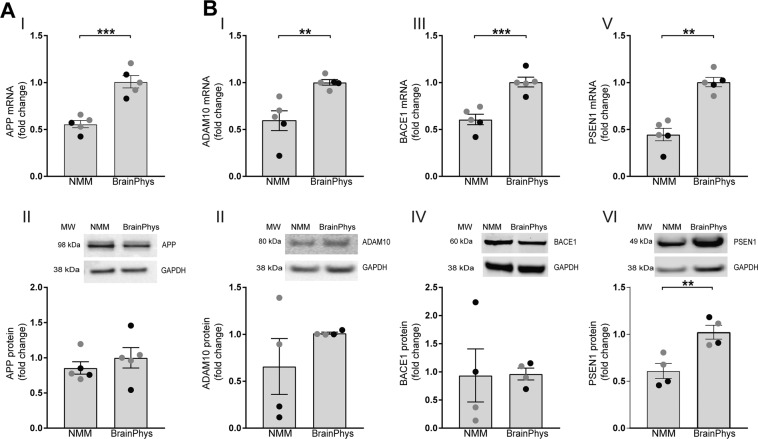
BrainPhys increases mRNA levels of APP and APP-cleaving enzymes. NPCs differentiated in either BrainPhys medium or NMM for up to 35 days and the gene- and protein expressions of APP and the APP-cleaving enzymes α-secretase (*ADAM10*), β-secretase (*BACE1*) and γ-secretase (*PSEN1*) investigated using qPCR and western blot, respectively. (**A**) *APP* mRNA levels (I) show a significant increase in BrainPhys-cultured cells compared with NMM, while no change in intracellular full-length APP protein levels (II) is observed. Mean values of five separate experiments on neurons from two different iPSC lines (Ctrl1 marked with grey circles and ChiPSC22 marked with black circles) were analysed with Student’s t-test. Bars represent mean +/− SEM. ***p ≤ 0.001. One representative blot out of three is shown. (**B**) *ADAM10* (a disintegrin and metalloproteinase domain-containing protein 10) mRNA levels (I) show a significant increase in BrainPhys-cultured cells compared with NMM, while no change in intracellular ADAM10 protein levels (II) is observed. *BACE1* (beta-site APP cleaving enzyme 1) mRNA levels (III) show a significant increase in BrainPhys-cultured cells compared with NMM, while no change in intracellular BACE1 protein levels (IV) is observed. Both *PSEN1* (presenilin1) mRNA levels (V) and protein levels (VI) increase significantly in the BrainPhys-cultured cells compared with NMM. Mean values of five (mRNA) or four (protein) separate experiments on neurons from two different iPSC lines (Ctrl1 marked with grey circles and ChiPSC22 marked with black circles) were analysed with Student’s t-test. Bars represent mean +/− SEM. **p ≤ 0.01, ***p ≤ 0.001. Full-length blots are presented in Supplementary Fig. 1.

To investigate if the increased secretion of APP cleavage products was linked to changed expression of APP or APP-cleaving enzymes, we measured mRNA and protein levels of APP, ADAM10 (α-secretase), BACE1 (β-secretase) and PSEN1 (γ-secretase) using qPCR and western blot, respectively. Culturing the cells in BrainPhys significantly increased *APP* mRNA levels (Fig. 7AI) whereas APP protein levels remained stable (Fig. 7AII). BrainPhys-cultured neurons displayed significantly increased mRNA levels of *ADAM10* (Fig. 7BI), *BACE1* (Fig. 7BIII) and *PSEN1* (Fig. 7BV). When analyzing protein levels of the secretases, no significant change was observed for ADAM10 (Fig. 7BII) or BACE1 (Fig. 7BIV) whereas a significant increase in PSEN1 was detected (Fig. 7BVI). Full-lengths blots of all proteins are presented in Supplementary Fig. 1.

### BrainPhys increases neuronal morphology and Aβ secretion in SH-SY5Y cells

**Figure 8:**
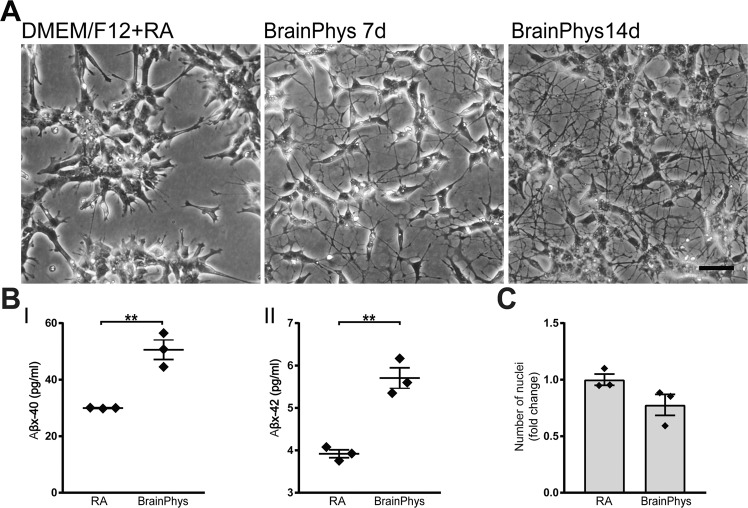
BrainPhys increases neuronal morphology and Aβ secretion in SH-SY5Y cells. SH-SY5Y neuroblastoma cells pre-differentiated with RA for five-seven days and thereafter seeded onto poly-l-ornithine- and laminin-coated plates and cultured either in standard medium (DMEM/F12) or BrainPhys for 7–14 days. (**A**) Phase contrast images of SH-SY5Y cells cultured in standard DMEM/F12 medium (left panel) and cells cultured in BrainPhys media for 7 days (middle panel) or 14 days (right panel) are shown. Increased neuronal morphology with longer and highly branched neurites is observed in BrainPhys-cultured SH-SY5Y cells already after 7 days (middle panel) and increase further with another 7 days (right panel) of BrainPhys culturing. Representative images from one out of three experiments are shown. Scale bar = 100 μM. (**B**) Concentrations of secreted Aβ peptides measured from cell-conditioned media of SH-SY5Y cells cultured in either DMEM/F12 supplemented with retinoic acid for 7 days or in BrainPhys media for 14 days. BrainPhys significantly increases the secretion of both (I) Aβ40 and (II) Aβ42 as compared to the standard medium (Aβ38 is below the lower limit of detection of the assay). Mean values of three separate experiments were analysed with Student’s t-test. **p ≤ 0.01. Bars represent mean +/− SEM. (**C**) The cell numbers from phase contrast images from three separate experiments were analysed with Student’s t-test. No significant differences in cell numbers were observed between NMM- and BrainPhys-cultured neurons. Bars represent mean +/− SEM presented as relative to NMM.

The neuroblastoma cell line SH-SY5Y is a commonly used neuronal cell model to study APP processing. The cell line is easy to culture and maintain, and can be differentiated with retinoic acid (RA) for one week to increase its neuron-like features^35^. However, Aβ secretion from these cells is low and close to the detection limit of most assays. To investigate if BrainPhys culturing could further increase the neuronal phenotype and secretion of APP cleavage products in this cell line, we cultured SH-SY5Y neuroblastoma cells in BrainPhys medium. SH-SY5Y cells were pre-differentiated with RA for seven days and thereafter seeded on poly-l-ornithine/laminin-coated wells and cultured in BrainPhys medium supplemented with RA for one or two weeks. Already after one week in BrainPhys, SH-SY5Y cells displayed a more definite neuronal morphology with more and longer neurites as compared to traditional culture conditions (Fig. 8A, left and middle panels). Increasing the BrainPhys culture time to two weeks (14 days) further increased this neuronal phenotype (Fig. 8A, right panel). Next, we investigated if the neuronal morphology would result in increased secretion of Aβ. Indeed, two weeks of BrainPhys culturing of SH-SY5Y cells resulted in more than 50% increase in secretion of Aβ40 and Aβ42 (Fig. 8BI–II), while Aβ38 was still under detection limit. To exclude that the increased secretion was due to increased cell number, cells were counted from phase contrast images of NMM and BrainPhys cultures. No significant difference in cell number was observed (Fig. 8C).

## Discussion

The BrainPhys medium was developed to provide a physiologically enriched, optimal environment for neuronal survival and improved synaptic activity *in vitro*^15^, while the effects on neuronal maturation were less in focus. To investigate if culture conditions favouring neuronal network formation and synaptic maturation would speed up neuronal differentiation, and if this in turn would affect APP processing and secretion of APP cleavage products, we compared cortical differentiation of NPCs in BrainPhys medium with the ordinary protocol using NMM^12^. We found that BrainPhys medium increased synaptic activity and markers of deep- but not upper-layer neurons, which coincided with increased secretion of APP cleavage products. In addition, we found that BrainPhys cultures had an increased Aβ38/42 ratio, suggesting a more non-amyloidogenic APP processing in this condition. This indicates that BrainPhys leads to neuronal maturation in shorter time during *in vitro* differentiation and that neuronal and synaptic maturation affects APP processing. However, extended culture in BrainPhys also resulted in the appearance and growth of non-neuronal cells types and all analyses had therefore to be performed at a time point before the complete maturation of NMM cultures^12^.

After less than 35 days of differentiation in BrainPhys, gene expression of the cortical deep-layer markers *TBR1* and *CTIP2* increased as compared to the NMM cultures, while markers of upper-layer neurons *SATB2, CUX1* and *BRN2* did not change. This suggests that neuronal differentiation in BrainPhys changes the cellular composition of the cultures towards an increased proportion of deep- over upper-layer neurons. In line with earlier findings that human neuroprogenitor cells cultured and matured in BrainPhys displayed dense neuronal networks and synaptically active neurons in a few weeks^15^, we observed that culturing cells in BrainPhys significantly increased the branching of neurites as compared to NMM already ten days after final plating. TBR1 and CTIP2 have been shown to play important regulatory roles for fasciculation and extension of sub-cortical axons, as well as for the establishment of neuronal connections and the increased expression of these markers could, in theory, contribute to the increased branching in BrainPhys cultures^36,37^. We also observed that the BrainPhys-cultured neurons displayed stronger staining of the neuronal microtubule proteins MAP2, tau and TUJ1, supporting that the neurons developed extensive neurite networks at an earlier time during differentiation.

The BrainPhys medium was designed to improve neuronal function and activity over what could be obtained using other cell culture media^15^. Here, we observed that BrainPhys gave rise to a stronger staining to markers for both pre-synapses (vGlut1, SV2) and post-synapses (PSD-95). In addition, it also increased the expression of presynaptic protein SNAP25 in mRNA level although protein levels of the protein remained unchanged. Furthermore, BrainPhys increased mRNA levels of *CAMK2B* and *ARC*. CAMK2B is a Ca^2+^/calmodulin-dependent protein known for its importance in long-term potentiation and synaptic plasticity^21,22^. *ARC* gene expression is commonly used as a neuronal-activity marker^23^, since *ARC* mRNA is induced by neuronal activity^24^ and accumulates in activated dendrites^38^.

Next, we used a multi-electrode array (MEA) system to analyze the effects of BrainPhys culturing on spontaneous activity. Although the neurons cultured in BrainPhys medium detached from the electrodes in MEA plates during long-term culturing, as was previously observed^39^, switching to BrainPhys for six to ten days was enough to increase both the number of spikes and the number of population bursts in mature neurons differentiated according to Shi *et al*.^12^. Taken together, our findings are in line with earlier findings^15^ and support the observation of increased neuronal networks and synaptic activity.

In addition to the observed increase in deep-layer cortical neuronal markers, astroglial markers were also increased in BrainPhys-cultured cells as compared to NMM. Astrocytes are important for neurons to form healthy and functional synapses^40^ and are known to regulate synaptogenesis through contact-mediated signaling and to control the structural synapse formation by secreting synaptogenic signals^28^. In the NMM protocol, a smaller number of astrocytes appear as the culture matures, usually after 50 days post-final plating^10^. Interestingly, culturing cells in BrainPhys medium increased the number of GFAP-positive cells after less than 35 days and, in addition, astrocytes positive for both GFAP and S100 were observed. Hence, increased neuronal activity and dense neuronal network in the BrainPhys-cultured cells could be an effect of the increased number of astrocytes in the cultures, which highlights the importance of proper characterization of cell cultures.

After characterizing cortical- and synaptic maturation of cells cultured in BrainPhys versus NMM media, we subsequently investigated how these culture conditions affected mRNA- and secretion levels of tau and NfL, two proteins involved in axonal assembly and synaptic maturation^41,42^. Tau secretion is high in the developing brain^43^, which may reflect neuronal plasticity during differentiation. Both tau and NfL are secreted under physiological conditions and the secretion increases with increased synaptic activity^44–46^. NfL was also recently suggested to be involved in synaptic activity as a modulator of neurotransmitter release^47,48^. Here, we found significantly increased expression and secretion of both tau and NfL in BrainPhys cultures compared with NMM. In NMM, detectable levels of NfL in the neuron-conditioned media usually appear as the cells mature, typically more than 40 days after the final plating. Consistently, here NfL was under or close to the detection limit of the assay in NMM, while BrainPhys culturing increased the secretion of NfL to clearly measurable levels.

Tau and NfL are also well-known markers for neurodegeneration and CSF tau concentrations reflect an AD-type neurodegeneration, while CSF NfL concentrations are increased across most neurodegenerative diseases^49,50^. To ensure that the increased secretion was not due to neuronal death, we measured cell death and viability in both conditions, but observed no difference between the two. This suggests that the increased levels of tau and NfL in the BrainPhys cultures were rather due to an active secretion process, possibly due to increased neuronal- and synaptic maturation and increased number of neurites.

Tau can exist in six different isoforms^51^, due to alternative splicing at two different sites. One of them is exclusion or inclusion of exon 10, resulting in three or four microtubule binding repeats (3R and 4R, respectively). 3R tau binds less tightly to microtubule^52^, which is necessary during development. 4R tau, where exon 10 is included, is absent in the fetal brain but increases dramatically during the prenatal- to adult period^31^. To determine the neuronal maturation state, we investigated presence of tau exon 10 in both conditions. No clear expression of tau exon 10 was observed in either NMM or BrainPhys cultures at this stage of differentiation. However, more than 200 days of culture in NMM was needed for this splice variant to appear. It is possible that exon 10 would appear earlier in BrainPhys than NMM with extended culture time in BrainPhys, but the overgrowth of other non-neuronal cell types with long-term BrainPhys cultures made this unfeasible.

Accumulation of Aβ peptides in amyloid plaques in the brain is a key feature of AD, and APP and its cleavage forms have been extensively studied in this regard. Still, the physiological roles of the different peptides resulting from APP processing are not completely understood. In order to investigate the effects of increased neuronal maturity and activation on APP processing, we measured the secretion of soluble APP and Aβ peptides in neuron-conditioned media and found that BrainPhys increased secretion of all APP cleavage products measured. Aβ secretion has been shown to correlate with synaptic activity^34,53–55^ and we have previously reported that secretion of long Aβ (Aβ38, Aβ40 and Aβ42) peptides into cell-conditioned media coincides with neuronal and synaptic maturation^10^. Thus, one possible reason for the increased secretion of APP cleavage products observed here is the increased neuronal characteristics of the BrainPhys-cultured cells. Moreover, we found that culturing SH-SY5Y cells in BrainPhys gave rise to a more neuronal phenotype, which also resulted in increased Aβ secretion. This shows that the increased neuronal maturation observed in BrainPhys cultures can be used as a model to investigate the effects of a neuronal phenotype on APP processing, as long as the cultures are properly characterized, and presents SH-SY5Y cells cultured in BrainPhys as a refined cell model to study APP processing *in vitro*.

To further investigate if the increased secretion of Aβ could be due to increased synaptic activity in the BrainPhys cultures, we blocked neuronal activity using TTX and measured Aβ secretion. Although neuronal activity was efficiently blocked, only a 20–30% reduction in Aβ secretion was observed. This suggests that synaptic activity affects Aβ secretion, but only to a certain extent and that other factors contribute. This is in line with our earlier findings that neurons start to secrete Aβ peptides around ten days after final plating in NMM before measurable neuronal activity is seen and networks have formed^10^.

Increased spikes and bursts due to an artificially applied electrical stimulus to neurons was previously shown to decrease the ratio of Aβ42 to Aβ40 through conformational changes in the γ-secretase subunit PSEN1^56^. We therefore investigated whether culturing neurons in BrainPhys would also affect the cleavage pattern of APP and the ratios of secreted Aβ peptides. Interestingly, we did not observe any change in the ratio of Aβ42 to Aβ40, but found the ratio of Aβ38 to both Aβ40 and Aβ42 to be significantly increased, indicating a relatively larger increase in secretion of Aβ38. Although it should be investigated in depth, BrainPhys-induced synaptic activity may cause conformational changes in PSEN1 favoring γ-cleavage of APP at Aβ amino acid 38 over 42^57^. This pattern is opposite to the one observed in familial AD patients carrying *PSEN1* mutations^58^, and could thus potentially protect from Aβ42-induced amyloidosis. Blocking synaptic activity did not significantly alter the individual peptide ratios in these culture conditions, suggesting that the increase in Aβ38 was due to other factors. Although neurons are considered the main Aβ-secreting cell type, glial cells also contribute to the Aβ load^8^. It is not determined which cleavage forms of Aβ that are mainly produced in glial cells, but increased Aβ38 secretion could in theory also be due to the larger number of glial cells in the BrainPhys cultures.

Increased secretion of APP cleavage products could be the result both of increased APP expression and/or increased APP processing. We found that the mRNA levels of *APP* were significantly increased in BrainPhys-cultured cells, whereas no significant differences were observed on protein level, indicating that BrainPhys culturing increases both APP synthesis and APP processing, resulting in stable levels of full-length APP protein in the cells. The basal APP protein levels varied between differentiations, for unknown reasons. How APP expression is regulated is not fully elucidated, but the suggested roles of APP in neuronal maturation, neurite outgrowth and synaptic formation indicate that APP expression is tightly regulated during these events^59^. As we have previously shown^10^, the processing of APP is also regulated throughout neuronal development and the relatively large variation in APP protein levels between differentiations could possibly be explained by slight differences in the timing of differentiation steps between differentiations and iPSC lines.

Similarly, mRNA levels of the main APP-cleaving enzymes *ADAM10* (α-secretase), *BACE1* (β-secretase) and *PSEN1* (γ-secretase) were all significantly increased in BrainPhys-cultured neurons, while only PSEN1 was significantly increased on protein level. Thus, the increase in PSEN1 protein could contribute to the increased Aβ secretion observed in BrainPhys cultures. Why the increased mRNA levels of ADAM10 and BACE1 were not reflected in protein levels remains to be investigated, but different post-transcriptional and -translational modifications along with protein degradation could contribute^60^. Synaptic activity also contributes to Aβ production through enhanced synaptic vesicle recycling, which causes more internalization of APP into endosomes. There, APP interacts with β- and γ-secretases resulting in increased production and secretion of sAPPβ and Aβ^54^. Similarly, active ADAM10 is enriched in synaptic vesicles^61^ and APP can be internalized in synaptic vesicles as a response to increased synaptic activity^62^. Thus, increased mRNA levels of α-, β- and γ-secretases in BrainPhys-cultured neurons could reflect an increased need for their activities due to enhanced internalization of APP in subcellular organelles and vesicles. The reason for increased sAPPα secretion (although less pronounced than the sAPPβ increase) could therefore be an enhanced interaction with APP in synaptic vesicles of BrainPhys-cultured cells.

In this study, we aimed at obtaining mature cortical neurons to study neural maturity and synaptic activity-dependent Aβ secretion in a shorter time period by using BrainPhys medium. We found that BrainPhys indeed did accelerate neuronal maturation and increase secretion of both sAPP and Aβ at an earlier time point in the differentiation protocol. However, with BrainPhys we also observed a shift in APP-processing favoring β-cleavage of APP in general and Aβ38 in particular.

In summary, BrainPhys culturing led to faster maturation of deep-layer cortical neurons, increased number of astrocytes and increased secretion of APP cleavage products, but with altered Aβ-peptide ratios. Further, long-term culturing in BrainPhys (more than 45 days) led to a substantial increase in non-neuronal cell types in the cultures. Thus, BrainPhys can be beneficial for *in vitro* studies focusing on the physiological role and turnover of APP, but proper characterization of the cellular composition of the cultures is crucial. Future studies will address potential links of increased number of deep-layer neurons and astrocytes, as well as the increased PSEN1 expression, to increased processing of APP in general and at Aβ amino acid 38 specifically.

## Material and Methods

### Cell culture

Three different human iPSC lines were used in this study, Ctrl1^63^, ChiPSC22 (Takara Bio Europe) and WTSIi015-A (EBiSC/Sigma Aldrich). The iPSCs were differentiated towards NPCs according to Shi *et al*.^12^, as previously described^10^. Briefly, starting with fully confluent iPSCs, neural induction was initiated by addition of neural maintenance media (NMM) supplemented with mouse Noggin-CF chimera (R&D Systems) and SB431542 (Stemgent) for 10 days. When a neuroepithelial layer had formed, the cells were detached with dispase (Thermo Fisher Scientific) and re-plated in NMM supplemented with FGF2 (Peprotech) on laminin-coated plates (Sigma Aldrich) for 4–5 days with medium changes every second day. To clean the cultures from unwanted cell types, the cell colonies were further passaged two to three times with dispase before day 25. On day 25, when neurogenesis occurred, the cells were dissociated with StemPro Accutase (Thermo Fisher Scientific) to obtain single cells. The cells were further passaged with StemPro Accutase for expansion before the final plating around day 35. At this point, the cells were counted using a NucleoCounter® NC-200™ (Chemometec) and re-plated at a confluency of 50 000 cells/cm^2^ onto poly-L-ornithine (Sigma Aldrich) and laminin-coated plates in NMM (see Supporting Table 1). The day after final plating, the medium was replaced either with fresh NMM or BrainPhys Neuronal Medium (see Supporting Table 2) and maintained for up to 55 days. Every second day, NMM was fully changed whereas half of the BrainPhys was replaced with fresh BrainPhys, according to the respective protocols.

SH-SY5Y neuroblastoma cells were cultured in (DMEM/F12, (Thermo Fisher Scientific) supplemented with 10% FBS (SigmaAldrich), 1% Glutamax and 1% Penicillin-Streptomycin. Cells were pre-differentiated with 10 μM retinoic acid (RA) (SigmaAldrich) for 7 days prior to seeding onto poly-L-ornithine- and laminin-coated plates. Thereafter, the cells were cultured in this medium or BrainPhys medium supplemented with 10 μM RA for 7–14 days with bi-weekly media changes. Cell-conditioned medium was collected after 48 hours of culturing.

### Immunocytochemistry (ICC)

Neurons on poly-L-ornithine and laminin-coated chamber slides (Ibidi) were fixed with cold 4% PFA (Histolab) for 10 min at room temperature and washed with TBS 3 × 5 min. For permeabilization, the samples were incubated with 0.3% Triton-X100 (Sigma Aldrich) in TBS for 15 min at room temperature and thereafter blocked in blocking buffer (5% donkey serum (SigmaAldrich), 0.3% Triton-X100 in TBS) for 1 hour at room temperature. Primary antibodies were diluted (see Supporting Table 3), in blocking buffer and the cells incubated over night at 4 °C or 1.5 hours in room temperature. Thereafter, the samples were washed with TBS and incubated with Alexa flour conjugated secondary antibodies (Thermo Fisher Scientific), diluted in blocking buffer (see Supporting Table 3 for antibodies and dilutions), for 1.5 hours at room temperature. The samples were washed 3 × 5 min with TBS and incubated with DAPI (1 μM; Thermo Fisher Scientific) after the first wash for 5 min at room temperature. The samples were washed once with water and then mounted using Ibidi mounting media (Ibidi) and kept at 4 °C until analysis.

### Confocal microscopy and image analysis

The samples were analyzed using an Eclipse Ti-E inverted confocal microscope with 60x objective and the NIS Element software (Nikon). Between Z-stacks 0.5 µm of distances were acquired. For large images, taken for GFAP and S100 staining, areas were randomly chosen and 5 images taken around those areas with 0.5 µm of distance between Z-stacks. From each sample, 10 images were randomly captured. Image analysis and number of cells and DAPI (nucleus) counting were performed using ImageJ within same setting for each figure^64^.

### Western blot

The cells were lysed in RIPA buffer, as described previously^10^ and protein determination was performed using the Pierce BCA protein assay kit according to the manufacturers’ protocol (Thermo Fisher Scientific). Equal amounts of protein for each sample was added into loading buffer and loaded into wells of a 4–12% Bis-Tris gel (NuPAGE™) and run with MES buffer (all from Thermo Fisher Scientific). Protein blot was performed onto a 0.2 µM nitrocellulose membrane (GE Healthcare) using semi-dry technique. After blotting, the membrane was blocked in 5% non-fat dry milk (BioRad laboratories) for 1 hour and incubated over night at 4 °C with primary antibodies diluted in blocking solution (see Supplementary Table 1 for a list of the antibodies used). The membrane was then washed with TBS-Tween (SigmaAldrich) and incubated with HRP-conjugated anti-mouse or anti-rabbit secondary antibodies (1:2000, Cell Signaling Technologies) in blocking solution for 1 hour at room temperature. For protein detection, SuperSignal West Dura Extended Duration Substrate (Thermo Fisher Scientific) was used and bands were visualized using ChemiDoc XRS+ (BioRad laboratories). To re-incubate with an HRP-conjugated glyceraldehyde-3-phosphate dehydrogenase (GAPDH) antibody (see Supplementary Table 1 for a list of the antibodies and concentrations used), the membranes were stripped using Restore stripping buffer (Thermo Fisher Scientific), blocked one hour with 5% non-fat dry milk and incubated with anti-GAPDH antibody in blocking solution for 1 hour at room temperature or with related primary antibody overnight at 4 °C. Band intensities were calculated using either Image Lab (BioRad laboratories) or Image Studio (LI-COR imaging systems) and correlated to GAPDH.

### RNA extraction and cDNA synthesis

Cells were lysed directly in the well by addition of 360 or 600 µL Buffer RLT supplemented with 4 mM dithiothreitol (Sigma-Aldrich). Total RNA was extracted and purified manually or on a QiaCube robotic workstation (QIAGEN), using the RNeasy Mini protocol according to manufacturer’s instructions. Total RNA concentrations were measured on a NanoDrop 2000/2000c spectrophotometer (Thermo Fisher Scientific) and diluted in RNase-free water to a final concentration of 25–50 ng/µL. cDNA was synthesized from 250–500 ng of total RNA using a High Capacity cDNA kit with RNase inhibitor (Applied Biosystems) in a total reaction volume of 20 µL and converted in a single-cycle reaction on a 2720 Thermal Cycler (Applied Biosystems); 25 °C for 10 min, 37 °C for 120 min and 85 °C for 5 min.

### Quantitative PCR

Quantitative PCR was performed using inventoried TaqMan Gene Expression Assays with FAM reporter dye in TaqMan Universal PCR Master Mix with UNG according to manufacturer’s instructions, in a total reaction volume of 25 µL. qPCR reactions were performed on Micro-Amp 96-well optical microtiter plates on a 7900HT Fast QPCR System (Thermo Fisher Scientific), using standard settings for Standard Curve qPCR. TaqMan Gene Expression Assays (all from Thermo Fisher Scientific) are listed in Supplementary 2. 2.5 ng cDNA was used in the PCR and all samples were run in duplicates. PCR results were analyzed with the SDS 2.3 software (Applied Biosystems) and the relative quantity of gene expression was determined using the ∆∆CT method^65^, with BrainPhys-cultured cells as the calibrator and average CT:s of RPL27, RPL30 and HPRT1 as endogenous reference.

### PCR of Tau exon-10 inclusion

Forward primer GTCCGTACTCCACCCAAGTC and reverse primer ATGAGCCACACTTGGAGGTC (ThermoFisher Scientific) were used with a HotStarTaq DNA Polymerase kit (SigmaAldrich) to amplify a region including exon 10 of the *MAPT* gene. The PCR was carried out in a 25-µL reaction consisting of 1x PCR buffer, 0.2 µM forward- and reverse primer, 0.2 mM of each dNTP, 0.65 Units/µL HotStar Taq DNA polymerase and 0.8 ng/µL cDNA from NMM- or BrainPhys-cultured neurons. The template was denatured at 95 °C for 15 min followed by a 2-cycle step-down amplification in three steps with annealing temperatures of 64 °C, 62 °C and 60 degrees, respectively. Amplification was then carried out by 30 cycles at 94 °C for 30 sec/58 °C for 30 sec/72 °C for 30 sec, followed by elongation of the product at 72 °C for 10 min.

### Multi-electrode array analysis of synaptic activity

Cells were differentiated towards cortical neurons according to Shi *et al*.^12^ for 35 days, then the cells were seeded on poly-L-ornithine (0.01%; Sigma Aldrich) and laminin-coated 60–6well MEAs plates (Multi Channel Systems MCS GmbH), in six independent culture chambers, with 9 electrodes in each chamber. NMM was replaced with BrainPhys medium ten days prior to analysis. MEA recordings were performed using a MEA2100-System (Multi Channels Systems MCS GmbH). Data acquisition and analysis were performed using the Multi-Channel Suite package (Multi Channels Systems MCS GmbH). The raw data was filtered at 200 Hz high pass (Butterworth, high pass filter) and further processed by a Spike Detector. The spike detection was performed using the Threshold (Falling edge) method, with the threshold set to 5x standard deviation. Spike counting was made from the electrodes which had 6 or more spikes. The pre-spike and post-spike durations were set to 1 ms and 2 ms, respectively. The burst detection was performed using the Maximum Interval Method with the minimal duration of a burst and minimal interval between bursts set to 50 ms and 100 ms, respectively. The maximum interval to start and end a burst was set to 50 ms and the minimum number of spikes in a burst was set to 5.

### Immunochemical quantification of sAPPα, sAPPβ and Aβ peptides

Medium was collected after 48 hours of incubation with cells, centrifuged at 400 × *g* for 5 minutes to remove cell debris and then frozen in −80 °C until analysis. Concentrations of sAPPα and sAPPβ in cell-conditioned media were measured using the MSD sAPPα/sAPPβ Multiplex Assay (Meso Scale Discovery) according to manufacturer’s instruction. Media concentrations of Aβ38, Aβ40 and Aβ42 were measured using the MSD Human (6E10) Abeta Triplex Assay (Meso Scale Discovery) as described by the manufacturer. For both assays, the detection limit was set to the value of the lowest standard point.

### Blocking of synaptic activity using tetrodotoxin

Tetrodotoxin (TTX, 6973.1 ROTH) was reconstituted in 0.1 M citrate buffer (sodium citrate dehydrate and citric acid) to a final concentration of 1 mM stock solution. Upon final plating in MEA plates, the cells were counted using a NucleoCounter® NC-200™ (Chemometec) and seeded at a confluency of 200 000 cells/cm^2^ to obtain equal cell numbers in all MEA wells. Ten days before treatment with vehicle or 1 µM TTX, the medium was switched from NMM to BrainPhys in all wells. After 24 hours of TTX/vehicle treatment, synaptic activity and Aβ secretion were assessed as described above.

### Immunochemical quantification of tau and NfL

Medium was collected after 48 hours of incubation with cells, centrifuged at 400 × *g* for 5 minutes to remove cell debris and then frozen in −80 °C until analysis. Total tau concentration in cell media was measured using INNOTEST® hTAU Ag ELISA (Fujirebio) according to the manufacturer’s instructions. The plate was mixed and the absorbance was quantified at 450 nm at a Multiskan FC (Thermo Scientific) within 15 min. NfL concentration in the cell media was measured using NF-light® (Neurofilament light) ELISA from UmanDiagnostics, according to the manufacturer’s instructions. The absorbance was quantified at 450 nm with 620 nm reference wavelength at a Multiskan FC (Thermo Scientific).

### Lactate dehydrogenase (LDH) assay

To compare the level of necrosis between cells cultured in NMM and BrainPhys, we performed an LDH activity assay, as described previously^66^. As a control for 0% viability, similarly aged cortical neurons were lysed with 1% Triton X-100 (Sigma Aldrich) (1% v/v to cell-conditioned media). The lysed cortical neurons (controls) were incubated at 37 °C for 30 min to release LDH. Then, the control and the samples were centrifuged at 360 × *g* for 5 min and the supernatants were collected. Freshly collected supernatants (350 μL) were analyzed at the Clinical Chemistry Laboratory at Sahlgrenska University Hospital, Gothenburg using the LDH activity test kit as described by the manufacturer (Roche Diagnostics Scandinavia AB, 03004732122) on the Cobas e501 module (Roche, Germany).

### Cell viability

To compare the viability between cells cultured in NMM and BrainPhys, we performed image cytometry on an Automated cell counter NucleoCounter® NC-200™ (Chemometec) according to manufacturer’s instructions. Using a Via1-Cassette™ (Chemometec), cells from 200 µl cell suspension were automatically stained with acridine orange and DAPI. The Via1-Cassette™ was inserted into the cell counter and the total and dead cell populations were determined with imaging cytometry.

### Statistical analysis

Mean values from separate experiments (n) were compared using Student’s two-tailed t-test (in Fig. 6B, secretion data after TTX, *p* values were extracted from one-tailed t-test, given that a decreased secretion was expected on the basis of earlier results^34^) and two-way ANOVA followed by Bonferroni’s multiple comparisons test. Each n represents neurons differentiated from different iPSC lines or neurons differentiated from the same iPSC line on separate occasions. Statistical significance was defined as p < 0.05. All statistical analyses were performed using GraphPad (Prism version 7.02 for Windows, GraphPad Software, La Jolla California USA, www.graphpad.com).

## Supplementary information

Supplementary information.
