# Author Correction: Accelerated neuronal and synaptic maturation by BrainPhys medium increases Aβ secretion and alters Aβ peptide ratios from iPSC-derived cortical neurons

**DOI:** 10.1038/s41598-020-61008-z

**Published:** 2020-02-28

**Authors:** Tugce Munise Satir, Faisal Hayat Nazir, Dzeneta Vizlin-Hodzic, Erik Hardselius, Kaj Blennow, Selina Wray, Henrik Zetterberg, Lotta Agholme, Petra Bergström

**Affiliations:** 1 Institute of Neuroscience and Physiology, Department of Psychiatry and Neurochemistry, the Sahlgrenska Academy at the University of Gothenburg, S-405 30 Gothenburg, Sweden; 2 Institute of Neuroscience and Physiology, Department of Psychiatry and Neurochemistry, the Sahlgrenska Academy at the University of Gothenburg, S-431 80 Mölndal, Sweden; 3 Clinical Neurochemistry Laboratory, Sahlgrenska University Hospital, S-431 80 Mölndal, Sweden; 4 Department of Neurodegenerative Disease, Institute of Neurology, University College London Queen Square, London, WC1N 3BG UK; 5 UK Dementia Research Institute at UCL, London, WC1E 6BT UK

Correction to: *Scientific Reports* 10.1038/s41598-020-57516-7, published online 17 January 2020

In the Supplementary Information file originally published with this Article, supplementary tables 1 and 2, detailing the Neuronal maintenance media ingredients and BrainPhys media ingredients respectively, were omitted. These errors have been corrected in the Supplementary Information that now accompanies the Article.

