# Supplementary material for: Accelerated neuronal and synaptic maturation by BrainPhys medium increases Aβ secretion and alters Aβ peptide ratios from iPSC-derived cortical neurons
Source: Sci Rep. 2020 Jan 17;10:601. doi: 10.1038/s41598-020-57516-7 (PMC6969066; doi:10.1038/s41598-020-57516-7)
Supplement: Supplementary file 1 — Supplementary information. [file 41598_2020_57516_MOESM1_ESM.pdf]

Accelerated neuronal and synaptic maturation by BrainPhys medium increases Aβ secretion and alters Aβ peptide ratios from iPSC-derived cortical neurons

Tugce Munise Satir[a*], Faisal Hayat Nazir[a,b], Dzeneta Vizlin-Hodzic[a], Erik Hardselius[b], Kaj Blennow[b,c], Selina Wray[d], Henrik Zetterberg[b, c, d, e], Lotta Agholme[a§], Petra Bergström[a§]

[a] Institute of Neuroscience and Physiology, Department of Psychiatry and Neurochemistry, the Sahlgrenska Academy at the University of Gothenburg, S-405 30, Gothenburg, Sweden

[b] Institute of Neuroscience and Physiology, Department of Psychiatry and Neurochemistry, the Sahlgrenska Academy at the University of Gothenburg, S-431 80, Mölndal, Sweden

[c] Clinical Neurochemistry Laboratory, Sahlgrenska University Hospital, S-431 80, Mölndal, Sweden

[d] Department of Neurodegenerative Disease, Institute of Neurology, University College London Queen Square, London, WC1N 3BG, UK

[e] UK Dementia Research Institute at UCL, London, WC1E 6BT, UK

[§] Equal contribution

∗ Corresponding author

Institute of Neuroscience and Physiology, Department of Psychiatry and Neurochemistry, The Sahlgrenska Academy at the University of Gothenburg, S-405 30, Gothenburg, Sweden.

Email address: tugce.munise.satir@gu.se (T.M. Satir)

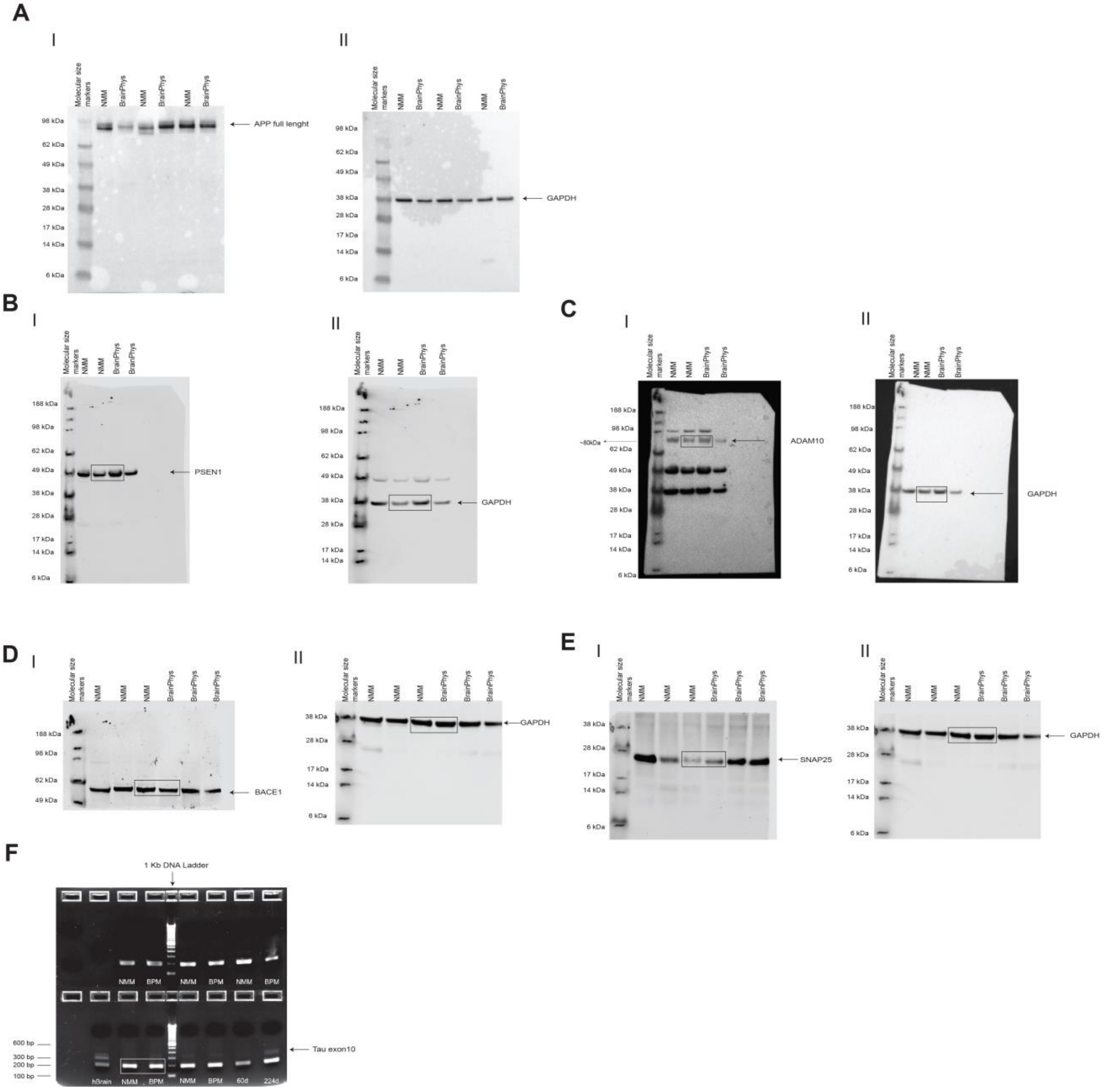

Supplementary figure 1. Representative images of whole membrane blots and agarose gel.

A) A representative blot of APP. The arrow points out the full-length APP band (~98 kDa). (II) A representative blot of the housekeeping protein GAPDH. The arrow points out the GAPDH band (~38 kDa). The black framed bands are the ones shown in the figures. The membrane is representative of three independent membranes.

B) A representative blot of PSEN1. The arrow points out the PSEN1 band (~49 kDa). (II) A representative blot of the housekeeping protein GAPDH. The arrow points out the

GAPDH band (~38 kDa). The black framed bands are the ones shown in the figures. The membrane is representative of two independent membranes.

C) A representative blot of ADAM10. The arrow points out the ADAM10 band (~80 kDa). (II) A representative blot of the housekeeping protein GAPDH. The arrow points out the GAPDH band (~38 kDa). The black framed bands are the ones shown in the figures. The membrane is representative of two independent membranes.

   *Due to lack of samples, membranes earlier incubated with PSEN1and GAPDH were stripped and used for ADAM10 and GAPDH. The extra bands (~50 and 38 kDa) on the ADAM10 blot are be therefore due to the previous antibody incubations of PSEN1 and GAPDH, however the ~98 kDa bands are unknown .

D) (I) A representative blot of BACE1. The arrow points out the BACE1 band (~60 kDa). (II) A representative blot of the housekeeping protein GAPDH. The arrow points out the GAPDH band (~38 kDa). The black framed bands are the ones shown in the figures. The membrane is representative of two independent membranes.

   *Due to lack of samples, membranes were cut into two pieces according to size of proteins of interest (below the 49 kDa). The same membrane was used for incubating SNAP25 and then stripped and used for GAPDH. The extra band on the GAPDH blot is therefore due to the previous SNAP25 antibody incubations.

E) (I) A representative blot of SNAP25. The arrow points out the SNAP25 band (~25 kDa). (II) A representative blot of the housekeeping protein GAPDH. The arrow points out the GAPDH band (~38 kDa). The black framed bands are the ones shown in the figures. The membrane is a representative of three independent membranes.

   *Due to lack of samples, membranes were cut into 2 pieces according to size of proteins of interest (below the 49 kDa). The same membrane was used for incubating SNAP25 and then stripped and used for GAPDH. The extra bands on the GAPDH blot is therefore due to the previous SNAP25 antibody incubations.

F) An image of the E-Gel precast agarose gel for tau exon10 PCR. The 1kb DNA ladder was used to distinguish the PCR-product sizes. The arrow points out the PCR product containing exon10. The white framed bands are the ones shown in the figures. (NMM= Neuronal Maintenance Media, BPM= BrainPhys Media, hBrain= human brain, 60d= 60 days old neurons cultured in NMM, 224d= 224 days old neurons cultured in NMM).

TABLES

Component	Catalogue numbers
DMEM/F12 L-glutamax (1:1)	31331-028 (Life Technologies)
Neurobasal media (1:1)	21103–049 (Life Technologies)
N2 supplement (1X)	17502-048 (Life Technologies)
B27 Supplement (1X)	17504–044 (Life Technologies)
MEM-NEAA (0.5 X)	11140-035 (Life Technologies)
Sodium Pyruvate (0.5 mM)	S8636 (SigmaAldrich)
Penicillin/Streptomycin (2500 U/ml)	SV30010 (Hyclone, GE)
β-mercaptoethanol (50 µM)	31350010 (Life Technologies)
Glutamax (100 µM)	25050-038 (Life Technologies)
Insulin (10 µg/ml)	I9278 (SigmaAldrich)

Table 1. Neuronal maintenance media ingredients

Component	Catalogue numbers
BrainPhys^{TM} Neuronal Medium	05790 (StemCell Technologies)
NeuroCult^{TM} SM1 Neuronal Supplement	05711 (StemCell Technologies)
N2 Supplement-A	07152 (StemCell Technologies)
BDNF (20 ng/ml)	450-02 (Peprotech)
GDNF (20 ng/ml)	450-10 (Peprotech)
Dibutyryl cAMP (1mM)	D0627 (SigmaAldrich)
L-ascorbic acid (200 nM)	A0278 (SigmaAldrich)
Penicillin/Streptomycin (2500 U/ml)	SV30010 (Hyclone, GE)

Table 2. BrainPhys media ingredients

Target Protein	Host	Company	Catalogue number	Dilution
**ADAM10**	Rabbit	Millipore	AB19026	WB 1:1000
**Anti-β-Amyloid, 1-16 Antibody**	Mouse	Biolegend	803002	WB 1:1000
**BACE1**	Mouse	SigmaAldrich	MAB5308	WB 1:1000
**GAPDH HRP conjugated**	Mouse	Novus biologicals	NB300-328H	WB 1:30000
**GFAP**	Chicken	Abcam	ab4674	ICC 1:1000
**MAP2**	Mouse	Abcam	ab11267	ICC 1:500
**Presenilin-1**	Rabbit	Biolegend	811101	WB 1:1000
**PSD-95 ab**	(Rabbit)	NeuroMab/UC DAVIS	ab18258	ICC 1:100
**S100**	Rabbit	Dako	Z0311	ICC 1:400
**SNAP25**	Rabbit	Sigma Aldrich	S9684	WB: :20000
**SV2 Synaptic vesicle glycoprotein 2A**	mouse	DSHB	SV2-c	ICC 1:500
**Tau antibody**	Chicken / IgY	Biorbyt	orb175815	ICC 1:1000
**Tuj1 (Beta-III-tubulin)**	Mouse	Abcam	ab14545	ICC 1:1000
**vGlut1**	Rabbit	Synaptic Systems	135303	ICC 1:750
**goat anti- mouse Alexa488**		Thermo Fisher Scientific	A11001	ICC 1:400
**goat anti-chicken Alexa488**		Thermo Fisher Scientific	A11056	ICC 1:400
**goat Anti-mouse Alexa568**		Thermo Fisher Scientific	ab175473	ICC 1:400
**goat anti-rabbit Alexa488**		Thermo Fisher Scientific	A11070	ICC 1:400
**goat Anti-rabbit Alexa568**		Thermo Fisher Scientific	A-11011	ICC 1:400

Table 3. Antibodies and concentrations

Gene Name	Company	Catalogue number
**Activity regulated cytoskeleton associated protein (ARC)**	Thermo Fisher Scientific	Hs01045540_g1
**ADAM metallopeptidase domain 10 (ADAM10)**	Thermo Fisher Scientific	Hs00153853_m1
**Amyloid beta precursor protein (APP)**	Thermo Fisher Scientific	Hs00169098_m1
**B-cell CLL/lymphoma 11B (CTIP2/BCL11B)**	Thermo Fisher Scientific	Hs01102259_m1
**Beta-secretase 1 (BACE1)**	Thermo Fisher Scientific	Hs01121195_m1
**Calcium/calmodulin dependent protein kinase II beta (CAMK2B)**	Thermo Fisher Scientific	Hs00365799_m1
**Cut like homeobox 1 (CUX1)**	Thermo Fisher Scientific	Hs00738851_m1
**Glial fibrillary acidic protein (GFAP)**	Thermo Fisher Scientific	Hs00909233_m1
**Hypoxanthine phosphoribosyl transferase 1 (HPRT1)**	Thermo Fisher Scientific	Hs02800695_m1
**Microtubule associated protein tau (MAPT)**	Thermo Fisher Scientific	Hs00902194_m1
**Neurofilament, light polypeptide (NFEL)**	Thermo Fisher Scientific	Hs00196245_m1
**Paired Box 6 (PAX6)**	Thermo Fisher Scientific	Hs00240871_m1
**POU class 3 homeobox 2 (BRN2)**	Thermo Fisher Scientific	Hs00271595_s1
**Presenilin 1 (PSEN1)**	Thermo Fisher Scientific	Hs00997789_m1
**Ribosomal protein L27 (RPL27)**	Thermo Fisher Scientific	Hs03044961_g1
**Ribosomal protein L30 (RPL30)**	Thermo Fisher Scientific	Hs00265497_m1
**Special AT-Rich Sequence-Binding Protein 2 (SATB2)**	Thermo Fisher Scientific	Hs00392652_m1
**Synaptosome associated protein 25 (SNAP25)**	Thermo Fisher Scientific	Hs00938957_m1
**T-box, brain 1 (TBR1)**	Thermo Fisher Scientific	Hs00232429_m1

Table 4. Primers used for qPCR
